# rs5911 and rs3842788 Genetic Polymorphism, Blood Stasis Syndrome, and Plasma TXB2 and hs-CRP Levels Are Associated with Aspirin Resistance in Chinese Chronic Stable Angina Patients

**DOI:** 10.1155/2017/9037094

**Published:** 2017-03-29

**Authors:** Mei Xue, Xuesong Yang, Lin Yang, Na Kou, Yu Miao, Mingming Wang, Junhua Ren, Quanli Zhao

**Affiliations:** ^1^Cardiovascular Center, Xiyuan Hospital, China Academy of Chinese Medical Sciences, Beijing, China; ^2^Department of Vascular Surgery, The First Hospital Affiliated to Shandong University of Traditional Chinese Medicine, Jinan, China; ^3^Physical Examination Center, Xiyuan Hospital, China Academy of Chinese Medical Sciences, Beijing, China

## Abstract

The identification of single nucleotide polymorphisms (SNPs) related to aspirin resistance (AR) is of great significance for the explanation why some individuals demonstrate an incomplete response to aspirin and for optimizing the antiplatelet therapy strategy. The study was designed to investigate the possible associated genetic markers and clinical factors of AR for Chinese patients with chronic stable angina after PCI and to analyze the association between TXA2, PGI2, hs-CRP level, AR, and gene polymorphisms. Totally 207 chronic stable angina patients who received 100 mg maintenance dose daily of aspirin for more than 7 days were enrolled. The inhibition of platelets was assessed using light transmittance aggregometry. TXB2, 6-keto-PGF1*α*, and hs-CRP were measured by radioimmunoassay. Genotyping was performed using Taqman probe technique (rs5787 and rs5911) and gene sequencing technology (rs3842788). By using binary logistic regression analysis, the impact of clinical and genetic determinants on AR was evaluated. The prevalence of AR and aspirin semiresistance (ASR) was 3.86% and 20.76%, respectively, in Chinese chronic stable angina patients. rs5911 A/C and C/C versus A/A genotype (OR = 5.546, 95% CI = 1.812–11.404), rs3842788 A/G versus G/G genotype (OR = 8.358, 95% CI = 2.470–28.286), and blood stasis syndrome (BSS, OR = 10.220, 95% CI = 4.242–24.621) were associated with AR, but rs5787 variants were all homozygous of G/G genotype. Plasma TXB2 and hs-CRP increased significantly in AR and ASR group, while 6-keto-PGF1*α* showed no difference, and TXB2 level was significantly higher in carriers of the rs3842788 A/G genotype. According to our results, rs5911 and rs3842788 are proved to be specific genetic markers of AR in Chinese chronic stable angina patients for the first time, and BSS was also proved to be a remarkable determinant for AR. The AR and ASR patients were with increased plasma TXB2 and hs-CRP levels, and the TXB2 level was influenced by the variation of rs3842788 genotype.

## 1. Introduction

Aspirin therapy is the cornerstone for the primary and secondary prevention of cardiovascular diseases, including stable angina pectoris and acute coronary syndrome (ACS) [1]. Despite adequate aspirin therapy, many patients still experience cardiovascular events, the phenomenon named as aspirin resistance (AR) [2]. Genetic variation may affect patients' response to drugs, so there are more and more researches focused on the identification of AR related gene polymorphisms. Since aspirin irreversibly inhibits platelet cyclooxygenase- (COX-) 1 to exhibit antiplatelet effect, the COX-1 gene polymorphisms are one of the foci. Maree et al. reported that genetic variability in COX-1 [A-842G, C22T (R8W), G128A (Q41Q, rs3842788), C644A (G213G), and C714A (L237M)] appears to modulate aspirin response in Irish patients (*n* = 144) with cardiovascular disease [3]. According to the results from International HapMap Project (http://hapmap.ncbi.nlm.nih.gov/) the mutation rates of COX-1 gene polymorphisms, except rs3842788, are very low in Han Chinese, even zero in C22T (R8W) and C714A (L237M). rs5787 variants in COX-1 exert the largest functional effects on decreasing the antiplatelet effectiveness of aspirin among four single nucleotide polymorphisms (SNPs) in vitro, with evidence for impaired interactions with a COX substrate and inhibitors [4]. But there is little known about the association of rs5787, rs3842788, and AR in Chinese stable pectoris patients. Another possible mechanism that may be involved in AR is the final pathway of platelet activation at the platelet glycoprotein (GP) IIb/IIIa receptor [5, 6]. Kranzhofer and Ruef reported that GP IIIa (PlA) polymorphism is related to AR in Turkish patients with intracoronary stent restenosis, while another research that enrolled Germany patients with coronary artery disease (CAD) showed AR is unrelated to differences in the PlA1/A2 single nucleotide polymorphism [7]. Our previous studies showed there is only PlA1/A1 allele of GP IIIa expressed in 251 Chinese patients, while the human platelet antigen- (HPA-) 3 polymorphism (rs5911) in GP IIb is proved to be an independent risk factor of CAD with the mutation of 31% [8, 9]. As compared to rs5911 C/C homozygotes, individuals with the rs5911 A/C genotype showed significantly increased inhibition of platelet aggregation in healthy Chinese male volunteers [10]. Since gene variants influence the therapeutic efficacy of drugs in a race-specific manner, it is very important to find specific genetic markers of AR in Chinese people for predicting patients' response to aspirin and optimizing the antiplatelet therapy strategy. Therefore, in the present study, we analyzed the association between SNPs of rs5787, rs3842788, and rs5911 in COX-1 and GPIIb/IIIa with AR in Chinese stable pectoris patients.

Prostaglandin I2 (PGI2) counteracts the vasoconstrictor and platelet aggregation effects of thromboxane A2 (TXA2), which maintains an important balance in cardiovascular homeostasis [11]. Inactivation of platelet COX-1 by aspirin leads to long-lasting suppression of TXA2 production and TXA2-mediated platelet activation and aggregation [12]. Doroszko et al. reported thromboxane in AR subjects was greater both at baseline and following aspirin therapy in a young healthy population (102 men aged 18–40) [13]. But the association between TXA2, PGI2 level, related gene polymorphisms, and AR in CAD patients remains unknown. The influence of high-sensitive C-reactive protein (hs-CRP), one of the cardiovascular risk markers, on aspirin response is still debated. Larsen et al. reported that increased levels of hs-CRP were associated with augmented platelet reactivity in stable high-risk CAD patients receiving aspirin 75 mg daily [14], while Raghavan et al. reported that aspirin exhibited no significant dose-dependent effect on hs-CRP in patients with type 2 diabetes [15].

Studies have shown that platelet activation, thrombosis, and restenosis after coronary stenting are closely related to blood stasis syndrome [8, 9]. However, little is known about the association of AR and blood stasis syndrome, especially lack of relatively large-scale study after adjustment of confounding factors with logistic regression analysis. Therefore, the present study was performed to investigate the possible associated genetic markers of AR for Chinese patients with chronic stable angina after percutaneous coronary intervention (PCI), analyze the association between TXA2, PGI2, hs-CRP level, BSS, AR, and gene polymorphisms, and investigate the independent risk factors of AR using binary logistic regression method.

## 2. Materials and Methods

### 2.1. Patients

This study was approved by the institutional Ethics Committee of China Academy of Chinese Medical Sciences, and all subjects gave written informed consent before participating. The trial is registered with ChiCTR-OCC-12002415 (Chinese Clinical Trial Register). All subjects enrolled were chronic stable angina patients with coronary angiography and showed stenosis ≥50% in at least one main coronary artery or previous myocardial infarction [16], who were 35–75 years old and have been taking 100 mg maintenance dose daily of aspirin for more than 7 days. Exclusion criteria included (1) family or personal history of bleeding disorders, (2) total platelet count <100 × 10^9^/L, or >450 × 10^9^/L, (3) hemoglobin < 90 g/L, (4) taking other antiplatelet, anticoagulant drugs or nonsteroidal antiinflammatory drugs within 30 days before test, (5) history of trauma or surgery within 2 weeks before enrollment, (6) severe primary diseases like renal insufficiency, liver dysfunction, hematopoietic system diseases, mental disorder, or malignant tumor, and (7) female in pregnancy or lactation period [17]. Classification of CHD syndrome was made according to the “Criterion of Syndrome Differentiation for CHD” by Cardiovascular Specialty Committee, China Association of Integrative Medicine [18]. For stasis syndrome differentiation and scores, refer to the “diagnostic criteria of blood stasis syndrome (BSS)” [19, 20] (Table 1).

### 2.2. Platelet Aggregation Testing

Platelet aggregation rate was assayed using light transmittance aggregometry (Platelet Aggregation Instrument, LBYNJ2, Beijing Lipusheng Co., China) with diphosphate adenosine (ADP, 10 *μ*M, Chrono-log Co., Havertown, USA) and arachidonic acid (AA, 0.5 mg/mL, Chrono-log Co., Havertown, USA) as inducers. AR is defined as ADP-induced platelet aggregation rate ≥70% and AA-induced platelet aggregation rate ≥ 20%, and aspirin semiresistance (ASR) is defined when one of the results was observed [21]. All of these assays were carried out as described previously [17].

### 2.3. TXB2, 6-Keto-PGF1*α*, and hs-CRP Detection

The TXA2- and PGI2-stable metabolites (TXB2 and 6-keto-PGF1*α*, resp.) [22], as well as hs-CRP (purchased from Huaying Biotechnology Institute, Beijing, China), were detected with radioimmunoassay (r-911, Industrial Corporation of China University of Science and Technology) according to the manufacturer's instructions.

### 2.4. Single Nucleotide Polymorphism (SNP) Selection and Genotyping

rs5787, rs3842788, and rs5911 were selected based on results from the International HapMap Project (http://hapmap.ncbi.nlm.nih.gov/) and previous investigations [9] (Table 2). Genotyping was performed using Taqman probe technique (rs5787 and rs5911) and gene sequencing technology (rs3842788) on an ABI PRISM 7900 HT Fast Real-Time Instrument (Applied Biosystems, Foster City, CA) and an ABI PRISM 3730 DNA Sequencer (Applied Biosystems, Foster City, CA, USA), respectively, as has been described [17].

### 2.5. Statistical Analysis

All statistical analyses were performed using SPSS version 15.0, and *P* value of less than 0.05 was considered statistically significant. Continuous variables were expressed as means ± standard deviation (SD), and one-way analysis of variance (ANOVA) was carried out for the comparison of means. Categorical data were described by frequency tables, percentage, or constituent ratio and compared using Chi-square test. Chi-square test or Fisher's exact test was used for testing whether the possible deviation of genotype distribution was consistent with Hardy-Weinberg equilibrium. A binary logistic regression model was used to identify independently associated clinical and genetic factors which significantly determine early AR in chronic stable angina patients.

## 3. Results

### 3.1. Baseline Characteristics of Study Participants and the Frequency of AR

Among 207 patients recruited in the present study, 8 (3.86%) patients were classified as AR, 43 (20.77%) as ASR, and the remaining 156 (75.36%) as aspirin sensitive (ASS) patients. AA-induced ASR occurred in 2.90% (6/207) patients and ADP-induced ASR in 17.87% (37/207) patients. The data of AR patients in combination with ASR patients were compared with that of ASS patients. There was no significance between the AR + ASR group and ASS group regarding differences in age, body mass index (BMI), underlying diseases (myocardial infarction history, dyslipidemia, and diabetes), being current smoker, drinking history, statins medication history, and serum lipid levels (*P* > 0.05) (Table 3). Notably, AR + ASR patients had a higher proportion of postmenopausal females than ASS patients (60.8% versus 39.7%, *P* < 0.05), whereas there were more males in the ASS group (60.3% versus 37.3%, *P* < 0.05). AR + ASR group also had more patients with hypertension (80.4% versus 63.5%, *P* < 0.05) and longer duration of CAD (7.94 ± 7.89 versus 5.79 ± 5.75, *P* < 0.05) when compared to ASS group.

### 3.2. Distribution of rs5787, rs3842788, and rs5911 Genotypes in AR + ASR and ASS Patients

Among the 207 enrolled patients, 76 patients were A/A homozygous (36.71%), 98 were A/C heterozygous (47.34%), and 33 were C/C homozygous (15.94%) of rs5911 gene polymorphism, which was in accordance with Hardy-Weinberg equilibrium. More patients carrying at least one C allele of rs5911 gene polymorphism were observed in AR + ASR group compared to those in ASS group (80.4% versus 57.7%, *P* < 0.01). Concerning rs3842788 gene polymorphism, 188 were homozygous with G/G genotype (90.8%) and 19 were heterozygous with A/G genotype (9.2%), but no patients with A/A genotype were observed in the present study. Patients with A/G genotype of rs3842788 were more susceptible to AR or ASR compared to G/G genotype (17.6% versus 6.4%, *P* < 0.05). All patients enrolled in the study were homozygous of G/G genotype in regard to rs5787 gene polymorphism. The genotype frequency distributions of rs5911 and rs3842788 in AR + ASR and ASS groups were presented in Tables 4 and 5.

### 3.3. Distribution of BSS in AR + ASR and ASS Patient

46 patients were with BSS among the 156 ASS patients, while 36 were found in 51 AR/ASR patients. The proportion of BSS in AR/ASR patients was significantly higher than that in ASS patients (70.6% versus 29.5%, *P* < 0.05, Table 6).

### 3.4. Binary Logistic Regression Analysis for Development of AR with Adjusted Age, Gender, and BMI

To explore the impact of risk factors on ASS and AR/ASR patients, binary logistic regression analysis adjusted by age, gender, and BMI is shown in Table 7. More patients with hypertension and longer duration of CAD were found in AR + ASR group compared to ASS group when analyzed using ANOVA or Chi-square test, but during binary logistic regression analysis, they were excluded as nonsignificant variables after adjusting for age, gender, and BMI. The A/C and C/C genotypes carriers of rs5911 were associated with an increased risk of AR than A/A homozygous carriers (OR = 5.546, 95% CI = 1.812–11.404, and *P* = 0.001). For the rs3842788 polymorphism, the A/G genotype significantly increased odds for AR development compared to G/G homozygous carriers (OR = 8.358, 95% CI = 2.470–28.286, and *P* = 0.001).

Female gender was found with an increased risk compared to male gender by binary logistic regression (OR = 5.269, 95% CI = 2.257–12.299.286, and *P* < 0.001), which may contribute to a higher proportion of postmenopausal females in AR + ASR patients than that in ASS patients (60.8% versus 39.7%, *P* < 0.05). Patients with BSS had significantly increased odds for AR (OR = 10.220, 95% CI = 4.242–24.621, and *P* < 0.001), which indicated that BSS was an important independent risk factor for AR.

### 3.5. The Association of Plasma TXB2, 6-Keto-PGF1*α*, and hs-CRP Levels and AR and Gene Polymorphisms

**Figure 1:**
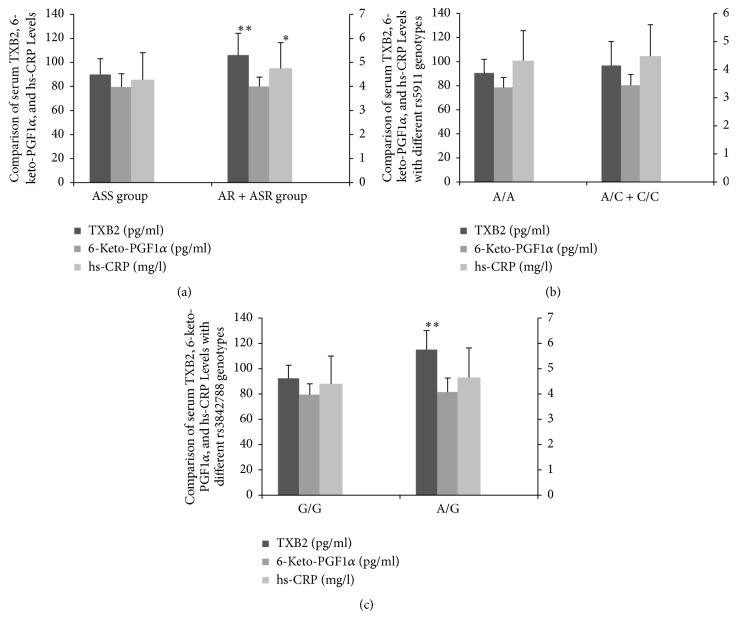
The association of plasma TXB2, 6-keto-PGF1*α*, hs-CRP Levels, AR, and gene polymorphisms. (a) Comparison of plasma TXB2, 6-keto-PGF1*α*, and hs-CRP Levels; (b) comparison of plasma TXB2, 6-keto-PGF1*α*, and hs-CRP levels with different rs5911 genotypes; (c) comparison of plasma TXB2, 6-keto-PGF1*α*, and hs-CRP Levels with different rs3842788 genotypes. ^*∗*^*P* < 0.05, compared with ASS group; ^*∗∗*^*P* < 0.01, compared with ASS group. The primary *y*-axis represented the concentration of TXB2 and 6-keto-PGF1*α*, and the secondary *y*-axis represented the concentration of hs-CRP.

Compared to ASS group, plasma TXB2 and hs-CRP levels increased significantly in AR + ASR group (*P* < 0.01), while no significant difference was present in plasma level of 6-keto-PGF1*α* (*P* > 0.05, Figure 1). The plasma TXB2, 6-keto-PGF1*α*, and hs-CRP levels were not different in patients with rs5911 A/A or A/C + C/C genotypes (*P* > 0.05). TXB2 level was significantly higher in carriers of the rs3842788 A/G genotype than in G/G carriers (*P* < 0.05). However, there was no difference in 6-keto-PGF1*α* and hs-CRP levels with different genotypes of rs3842788 (*P* > 0.05).

## 4. Discussion

Previous investigations reported that 6.2% to 57% patients were identified as AR or aspirin nonresponsiveness depending on the method used for detection and the different population studied [23–25]. In the present study, the prevalence of AR and ASR was 3.86% and 20.76%, respectively, assayed using turbidimetry in Chinese chronic stable angina patients, which was obviously lower than that found in other studies. Interestingly, the frequency of ADP-induced ASR was significantly higher than that of AA-induced one (17.87% versus 2.90%), which might be attributable to the selective inhibition of aspirin on AA metabolism.

Many cardiovascular risk factors have been reported to be associated with AR, for example, diabetes, lipid disorders, and history of being current smoker [26, 27]. No association was found between AR and myocardial infarction history, dyslipidemia, diabetes, being current smoker, drinking history, and statins medication history in the Chinese chronic stable angina patients. Our results also showed that AR is more common in patients with hypertension and longer duration of CAD and women when analyzed using ANOVA or Chi-square test. However, during binary logistic regression analysis, hypertension and longer duration of CAD were proved to have no influence on the occurrence of AR after adjusting for age, gender, and BMI. Female gender was proved to have an increased risk for AR compared to male gender by binary logistic regression. Since higher proportion of postmenopausal females was found in AR + ASR group compared to that in the ASS group, it may be the reason for women to be more likely stricken with AR.

Individualized medicine promises to take individual genome variations into account to optimize disease treatment [28]. Since aspirin is the most widely used antiplatelet drug, the identification of SNPs related to AR is of great significance for the explanation why some individuals demonstrate an incomplete response to aspirin and for the choice of antiplatelet drugs. COX-1 gene polymorphism may affect the enzyme expression and function and its interaction with aspirin [3]. Hundreds of COX-1 SNPs have been reported and many of them are rare in different ethnic groups. According to the International HapMap Project and previous investigation results, rs5787 and rs3842788 were investigated in the present study. And it was reported for the first time that rs5787 variants were all homozygous of G/G genotype in Chinese patients, with an extremely low mutation rate that could not be used as a marker of AR. 90.8% of patients were homozygous with G/G genotype and 9.2% with A/G genotype of rs3842788 in the enrolled patients, and the mutant frequency of rs3842788 was slightly lower in Chinese chronic stable angina patients compared with the results from HapMap Project-HCB (http://www.ncbi.nlm.nih.gov/projects/SNP/snp_viewTable.cgi?pop=1410, 45 unrelated Han Chinese in Beijing, 9.2% versus 14.3%).

rs5911, with higher mutation rate in Chinese population than the other SNPs in GP IIb, was proved to be a specific marker of AR for the first time. The patients with mutant genotype of rs5911 carrying at least one C allele were proved to be more susceptible to AR or ASR. More chronic stable angina patients with A/G genotype in rs3842788 were AR or ASR when compared to G/G genotype. C allele carriers of rs5911 and A/G genotype carriers of rs3842788 were independently associated with 4.5-fold and 8.3-fold higher incidence of AR when analyzed by binary logistic regression. Since the genetic variations of rs5911 in GP IIb may not directly modulate aspirin pharmacokinetics and pharmacodynamics as the SNPs in COX-1, its underlining mechanisms need further study.

Previous studies confirmed that BSS is closely related to platelet activation and thrombosis, which is the most common TCM syndrome in coronary heart disease [8, 9]. In the present study, it is noteworthy that the blood stasis syndrome was proved to be a remarkable determinant for AR, with as high as 10.2-fold incidence of AR compared to non-blood stasis syndrome. Therefore, treatment focused on blood stasis syndrome may improve AR, which provided a new approach for clinical prevention and treatment of AR. Our previous study confirmed that Xuefuzhuyu oral liquid, derived from the classic recipe Xuefuzhuyu decoction, could effectively improve blood stasis syndrome and AR by inhibiting ADP-induced platelet aggregation [17], which provides a broad prospect for the clinical application of Chinese medicine for promoting blood circulation and removing blood stasis in prevention of AR.

TXB2, mainly derived from platelets COX-1 pathway, is a potent stimulus for platelet aggregation reflecting the inhibition of COX-1 and COX-2 enzymes by aspirin upon a maximal platelet activation during clotting, while PGI2, mainly derived from endothelial cells, serves as a potent inhibitor of platelet aggregation [29, 30]. In our present study, plasma TXB2 increased significantly in AR and ASR patients despite optimal compliance, while no significant difference was present in plasma level of 6-keto-PGF1*α* (the PGI2-stable metabolite), indicating TXB2 could be used as a specific marker to evaluate COX-1 inhibition and patients' response to low-dose aspirin. And plasma TXB2 generation was also proved to be related to the variation of rs3842788 genotype in COX-1, which in part explains the heterogeneity in the way patients respond to aspirin. Plasma hs-CRP level also increased significantly in AR and ASR group, which was in accordance with the results from Larsen et al. [14], who reported that increased levels of hs-CRP were associated with augmented platelet reactivity in Denmark stable high-risk CAD patients receiving aspirin as mono antiplatelet therapy, but no genetic variations in COX-1 or GPIIb were found to be related to hs-CRP level.

## 5. Conclusions

It is proved for the first time that rs5911 and rs3842788 are independently associated with AR in Chinese chronic stable angina patients, with an increased risk as high as 4.5-fold and 8.3-fold, respectively. Blood stasis syndrome was also proved to be a remarkable determinant for AR, with as high as 10.2-fold incidence of AR compared to non-blood stasis syndrome. Significantly increased TXB2 and hs-CRP levels were demonstrated in AR and ASR patients and the TXB2 level was also proved to be related to the variation of rs3842788 genotype. Therefore, rs5911 and rs3842788 could be used as specific genetic markers of AR in Chinese population for optimizing the antiplatelet therapy strategy in clinic, and BSS could also be used as potential predictor for AR. More rigorous randomized controlled trials should be considered to further clarify the association between the polymorphisms and AR because of the relatively small study size in our present study.

## Figures and Tables

**Table 1:** Diagnostic criteria of blood stasis syndrome for coronary heart disease.

Classification	Contents	Score
Primary indicators (PIs)	(1) Chest pain of fixed location	10
	(2) Dark or purple tongue	10
	(3) Petechia or ecchymosis of tongue	10
	(4) Coronary angiography showed at least one coronary artery stenosis of 75%	9
	(5) Ultrasonic cardiogram or coronary angiogram (CAG) shows coronary thrombosis or ventricular mural thrombus	8

Secondary indicators (SIs)	(1) Chest pain aggravated at night	6
	(2) Dark purple lips or gum	
	(3) Varicose or dark purple sublingual veins	7
	(4) CAG showed at least one coronary artery stenosis of 50% but <75%	7
	(5) Shortened activated partial thromboplastin time (APTT) or prothrombin time (PT)	6

Assisted indicators (AIs)	(1) Darkish complexion	5
	(2) Uneven pulse	4
	(3) Severe vascular calcification or coronary diffuse lesion in CAG or computed tomographyangiography (CTA)	3
	(4) Elevated serum fibrinogen	3

*Notes*. (1) As for scientific research, CHD should be generally diagnosed by CAG showing at least one coronary artery stenosis of 50%; (2) BSS should be diagnosed with scores 19, and specific scores can be used to evaluate the severity of BSS for CHD; (3) the diagnosis of BSS for CHD should consist of at least one macroindicator (symptom or body sign) of PIs or SIs rather than diagnosing only depending on physical and chemical indicators.

**Table 2:** COX-1 and GPIIB SNPs.

	Region	Contig position	mRNA position	dbSNP rs # cluster id	RefSNP allele	Protein residue	Codon position	Amino acid position
COX-1	exon_4	32462028	458	rs5787	G	Arg [R]	2	108
					A	Gln [Q]	2	108
	exon_3	32461411	258	rs3842788	A	Gln [Q]	3	41
					G	Gln [Q]	3	41
GPIIb	exon_26	1106870	2653	rs5911	A	Ile [I]	2	874
					C	Ser [S]	2	874

*Notes*. dbSNP: single nucleotide polymorphism database; RefSNP: reference single nucleotide polymorphism.

**Table 3:** Baseline characteristics of study participants.

	ASS group (*n* = 156)	AR + ASR group (*n* = 51)	*P* value
Age (years), mean ± SD	61.8 ± 8.4	64.2 ± 8.2	0.076
Gender, *n* (%) male	94 (60.3)	19 (37.3)	0.004
Postmenopausal female, *n* (%)	62 (39.7)	31 (60.8)	0.018
BMI (kg/m^2^), mean ± SD	26.3 ± 3.9	26.0 ± 4.6	0.622
Duration of CAD (years), mean ± SD	5.79 ± 5.75	7.94 ± 7.89	0.037
Myocardial infarction history, *n* (%)	16 (10.3)	9 (17.6)	0.160
Hypertension, *n* (%)	99 (63.5)	41 (80.4)	0.025
Dyslipidemia, *n* (%)	101 (64.7)	37 (72.5)	0.305
Diabetes, *n* (%)	54 (34.6)	24 (47.1)	0.111
Current smoker, *n* (%)	23 (14.7)	6 (11.8)	0.595
Drinking history, *n* (%)	28 (17.9)	5 (9.8)	0.168
Statins, *n* (%)	88 (56.4)	36 (70.6)	0.073
TG (mmol/l), mean ± SD	1.59 ± 1.09	1.45 ± 0.66	0.390
TC (mmol/l), mean ± SD	4.60 ± 1.19	4.56 ± 1.03	0.846
LDL-c (mmol/l), mean ± SD	2.69 ± 1.04	2.74 ± 0.91	0.773
HDL-c (mmol/l), mean ± SD	1.06 ± 0.24	1.07 ± 0.23	0.810

*Notes*. BMI, body mass index; TG, triglyceride; TC, total cholesterol; HDL-c, high density lipoprotein cholesterol; LDL-c, low-density lipoprotein cholesterol.

**Table 4:** Distribution of rs5911 genotypes in AR + ASR and ASS groups.

Group	Genotype frequency, *n* (%)		*P* value
	A/A	A/C + C/C	
ASS	66 (42.3%)	90 (57.7%)	0.004
AR/ASR	10 (19.6%)	41 (80.4%)^*∗∗*^	

*Notes*. ^*∗∗*^*P* < 0.01, compared with ASS group.

**Table 5:** Distribution of rs3842788 genotypes in AR + ASR and ASS groups.

Group	Genotype frequency, *n* (%)		*P* value
	G/G	A/G	
ASS	146 (93.6%)	10 (6.4%)	0.016
AR/ASR	42 (82.4%)	9 (17.6%)^*∗*^	

^**∗**^ *P* < 0.05, compared with ASS group.

**Table 6:** Distribution of BSS in AR + ASR and ASS patients.

	BSS	NBSS	*P* value
ASS	46 (29.5%)	110 (70.5%)	0.000
AR/ASR	36 (70.6%)^*∗∗*^	15 (29.4%)	

^*∗∗*^ *P* < 0.01, compared with ASS group. NBSS: non-blood stasis syndrome.

**Table 7:** Binary logistic regression analysis for development of AR.

Covariates	*B*	SE	Wald	95.0% CI	*P*	Odds ratio (OR)
Age (years)	0.036	0.025	2.048	0.987–1.089	0.152	1.036
Gender (female)	1.662	0.433	14.762	2.257–12.299	0.000	5.269
BMI, kg/m^2^	−0.023	0.049	0.222	0.887–1.076	0.638	0.977
Duration of CAD (years)	0.022	0.034	0.438	0.957–1.093	0.508	1.023
Hypertension	−0.670	0.481	1.938	0.199–1.314	0.164	0.512
BSS	2.324	0.449	26.845	4.242–24.621	0.000	10.220
rs5911 (A/C + C/C versus A/A)	1.514	0.469	10.414	1.812–11.404	0.001	4.546
rs3842788 (A/G versus G/G)	2.123	0.622	11.652	2.470–28.286	0.001	8.358

*Notes*. *B*: beta coefficient; SE: standard error; Wald: Wald's statistics.
